# Convergent Regenerative Strategies in PM&R for Musculoskeletal and Hair Restoration: Integration of PRP, Exosomes, and Physical Modalities

**DOI:** 10.26502/aimr.0234

**Published:** 2026-02-19

**Authors:** Andre Aabedi, Devendra K. Agrawal

**Affiliations:** 1 Department of Translational Research, College of Osteopathic Medicine of the Pacific, Western University of Health Sciences, Pomona, California 91766 USA

**Keywords:** Exosomes, Hair follicle regeneration, Low-level laser therapy (LLLT, photobiomodulation), Mechanotransduction, Musculoskeletal healing, Physical modalities, Platelet-rich plasma (PRP), Regenerative medicine

## Abstract

Regenerative medicine has emerged as a transformative approach for both musculoskeletal disorders and hair follicle dysfunction by targeting shared biological mechanisms underlying tissue repair and renewal. Conditions such as tendinopathies, osteoarthritis, and alopecia contribute substantially to physical morbidity and psychosocial burden, while conventional therapies often provide limited or symptomatic relief. This Physical Medicine and Rehabilitation-centered review synthesized evidence on convergent regenerative pathways involved in musculoskeletal healing and hair follicle restoration, with a focused analysis of platelet-rich plasma, exosomes and cell-free biologics, and physical modalities, including low-level laser therapy and mechanotransduction. Across both tissue systems, these modalities modulate stem cell activity, angiogenesis, inflammatory signaling, and extracellular matrix remodeling through shared molecular pathways, including Wnt/β-catenin, TGF-β, IGF-1, PDGF, and VEGF signaling. Despite tissue-specific differences in cellular architecture and repair demands, overlapping regenerative mechanisms enable translational application of biologic, photo-biomodulatory, and mechanical therapies across orthopedic and dermatologic contexts. This review highlights clinical evidence, practical considerations, and regulatory challenges, while identifying gaps in standardization, dosing, and outcome measures. By framing hair follicle restoration and musculoskeletal healing within a unified regenerative paradigm, physical medicine and rehabilitation is positioned to bridge these traditionally distinct domains and advance biologically driven, minimally invasive therapies aimed at true tissue regeneration rather than symptom modulation alone.

## I. Introduction

Regenerative medicine represents a paradigm shift in treating musculoskeletal disorders and hair follicle dysfunction by addressing the fundamental limitations of conventional therapies through biologically driven tissue repair mechanisms. Musculoskeletal conditions such as tendinopathies and osteoarthritis contribute to substantial morbidity through chronic pain and functional impairment, while hair loss disorders including androgenetic alopecia and alopecia areata impose significant psychosocial burdens [1–2]. Conventional treatments for both categories often demonstrate limited efficacy, undesirable side effects, or require invasive interventions, necessitating the development of novel therapeutic strategies [3].

This review synthesizes evidence on regenerative pathways common to musculoskeletal and hair follicle healing, focusing on modalities applicable to physical medicine and rehabilitation practice. The objective is to identify shared molecular mechanisms, examine clinical applications across both domains, and propose opportunities for translational innovation.

**Figure 1:**
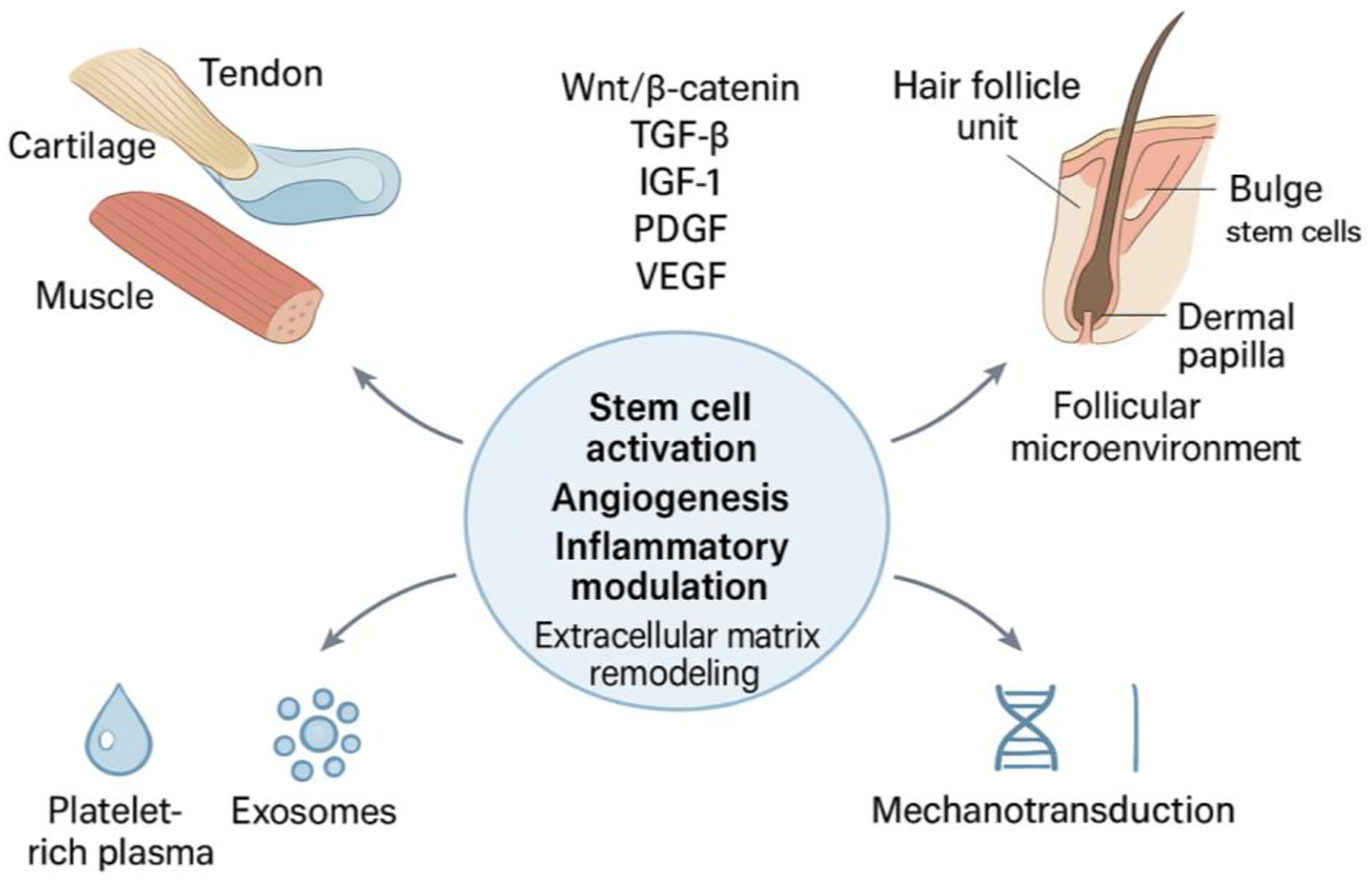
Shared regenerative modalities including platelet-rich plasma, exosomes, low-level laser therapy, and mechanotransduction converge on common molecular pathways that regulate stem cell activity, angiogenesis, inflammation, and extracellular matrix remodeling in both musculoskeletal tissues and hair follicles.

Four primary regenerative modalities form the framework of this analysis. Platelet-rich plasma (PRP), an autologous blood concentrate enriched with growth factors and cytokines, has demonstrated regenerative capacity in tendon, cartilage, bone, and hair follicle tissues [4–8]. Exosomes function as intercellular messengers that facilitate tissue regeneration across multiple systems including musculoskeletal and dermatologic structures [9–11]. Low-level laser therapy (LLLT) enhances cellular metabolism and tissue repair in both orthopedic rehabilitation and hair restoration contexts [1]. Mechanotransduction, which is the conversion of mechanical forces into biochemical signals, drives tissue adaptation and represents an increasingly recognized therapeutic mechanism in both musculoskeletal and follicular biology (Figure 1).

Through integrated analysis of these modalities, this review elucidates the convergent regenerative pathways and evidence-based clinical strategies that bridge musculoskeletal healing and hair follicle restoration within the scope of physical medicine and rehabilitation (PM&R).

## II. Shared Biological Foundations of Regeneration

Regenerative medicine in musculoskeletal and hair follicle systems operates through convergent biological principles: cellular proliferation and differentiation, extracellular matrix remodeling, angiogenesis, and inflammatory modulation. These processes are coordinated by growth factors, cytokines, and stem cell populations that respond to therapeutic interventions including platelet-rich plasma, exosomes, low-level laser therapy, and mechanotransduction.

Mesenchymal stem cells (MSCs) constitute the primary cellular mediators in both tissue systems, responding to biochemical and mechanical cues within specialized niches. PRP provides a concentrated growth factor milieu, including platelet derived growth factor (PDGF), vascular endothelial growth factor (VEGF), transforming growth factor beta (TGF-β), and insulin like growth factor 1 (IGF-1), that activates stem cell proliferation, guides differentiation, and promotes vascularization [6–8], [12–13]. Exosomes complement these effects through paracrine signaling, delivering microRNAs and proteins that regulate regenerative cascades [14].

Stem cell niches exhibit both commonalities and specialization. Musculoskeletal MSC populations reside in mechanically responsive environments that mediate injury repair and tissue homeostasis [8], [12]. Hair follicle stem cells, concentrated in the bulge region, drive cyclical regeneration with distinct epithelial and mesenchymal components [1],[15]. Despite functional differences, continuous homeostasis versus cyclical renewal, both niches respond to analogous molecular signals and growth factor stimulation.

Several key pathways mediate regeneration across both systems. Wnt/β-catenin signaling drives stem cell activation and lineage commitment, regulating dermal papilla function and anagen initiation in hair follicles while directing osteogenesis and chondrogenesis in musculoskeletal tissues [14]. TGF-β modulates the balance between regenerative repair and fibrotic responses, controlling inflammation and cellular differentiation in both contexts [8], [12]. The growth factors IGF-1, PDGF, and VEGF promote cellular proliferation, survival, and angiogenesis essential for both follicular cycling and musculoskeletal repair [5]. Precise inflammatory regulation proves critical, with PRP and exosomes facilitating transitions toward anti-inflammatory, pro-regenerative microenvironment [7].

Physical medicine modalities further engage these pathways. LLLT activates Wnt/β-catenin signaling and enhances mitochondrial function, stimulating regeneration in both tissue types [1]. Mechanotransduction through controlled mechanical loading triggers stem cell activity and growth factor secretion, amplifying endogenous repair mechanisms across musculoskeletal and follicular systems.

## III. Platelet-Rich Plasma (PRP)

Platelet rich plasma represents an autologous biological therapy employing convergent regenerative mechanisms across musculoskeletal and hair follicle systems. PRP contains concentrated platelets, growth factors (including VEGF, PDGF, and TGF-β), cytokines, and extracellular vesicles that collectively facilitate tissue repair through angiogenesis, immunomodulation, matrix synthesis, and cellular recruitment. These mechanisms support tendon, cartilage, and bone repair in musculoskeletal applications while promoting follicular stem cell activation and dermal papilla cell proliferation in hair restoration [4], [7], [16].

In musculoskeletal medicine, PRP has been applied to tendinopathies, osteoarthritis, and muscle injuries. The American Medical Society for Sports Medicine recognizes that PRP modulates inflammation and healing through rapid growth factor release, with the strongest clinical evidence supporting its use in tendinopathy and osteoarthritis [17]. PRP accelerates the wound healing cascade, enhances cellular recruitment, and facilitates matrix remodeling in musculoskeletal tissues. Evidence supports its efficacy particularly in chronic tendon injuries and mild to moderate osteoarthritis [6].

For hair restoration, PRP has demonstrated efficacy in treating androgenetic alopecia through activation of dermal papilla cells. Multiple randomized controlled trials and systematic reviews show that PRP increases hair count and density in androgenetic alopecia, with histologic confirmation of enhanced follicular proliferation and dermal papilla cell activation [2], [5], [18], [19], [20]. The mechanism involves promoting hair follicle stem cell proliferation, extending the anagen phase, and upregulating growth factors essential for folliculogenesis [1], [21]. Sonicated PRP preparations may further amplify stem cell activation and support de novo follicle regeneration [21].

Both musculoskeletal and hair follicle applications share core regenerative pathways, including growth factor mediated angiogenesis, immunomodulation, and matrix synthesis. However, tissue specific responses distinguish the two: musculoskeletal healing relies more heavily on fibrinolytic processes and mesenchymal stem cell recruitment, whereas hair follicle restoration depends primarily on follicular stem cell activation and dermal papilla signaling [16]. Preparation heterogeneity and the absence of standardized protocols remain significant challenges across both domains, underscoring the need for further research and consensus development [6].

## IV. Exosomes and Cell-Free Biologics

Exosomes and cell free biologics serve as key mediators of regenerative processes in both musculoskeletal and hair follicle systems, functioning primarily through paracrine signaling and the delivery of bioactive cargo including microRNAs, proteins, and lipids. These extracellular vesicles facilitate intercellular communication and regulate cell proliferation, migration, anti-apoptosis, angiogenesis, and immunomodulation. In musculoskeletal tissues, exosomes derived from stem cells or mesenchymal stromal cells enhance endogenous cell functions, promote tissue repair, and regulate inflammation by transferring specific microRNAs and growth factors that activate regenerative pathways [22–27]. Within the hair follicle, exosomes influence cycling and regeneration by stimulating dermal papilla cells and hair follicle stem cells, supporting angiogenesis, and modulating the local immune environment [28–31].

Preclinical evidence for the use of exosomes in musculoskeletal healing has been substantial, with studies demonstrating accelerated bone, cartilage, and tendon repair, reduced inflammation, and improved functional outcomes. Engineering strategies that load exosomes with therapeutic microRNAs or integrate them with biomaterials for sustained release have further enhanced their efficacy and tissue specificity [24]. Clinical translation remains in progress, with ongoing challenges related to standardization, targeting, and retention time [27].

In hair follicle restoration, exosomes derived from MSCs have shown efficacy in preclinical studies and early clinical trials for conditions such as androgenetic alopecia and nonscarring alopecias. These exosomes promote hair growth by activating dermal papilla cells, supporting stem cell function, and delivering microRNAs that regulate follicle cycling and immune response [28]. Pilot clinical trials have reported encouraging safety and feasibility data, though larger studies are necessary to confirm efficacy and optimize dosing protocols [30].

MicroRNA and paracrine signaling pathways represent critical links between musculoskeletal and hair follicle regeneration. Exosomes deliver microRNAs that modulate Wnt/β-catenin, TGF-β, and other signaling cascades essential for stem cell activation, angiogenesis, and tissue remodeling in both systems [29], [31]. Paracrine effects mediated by cytokines, chemokines, and growth factors support repair and homeostasis across tissue types [31].

**Figure 2:**
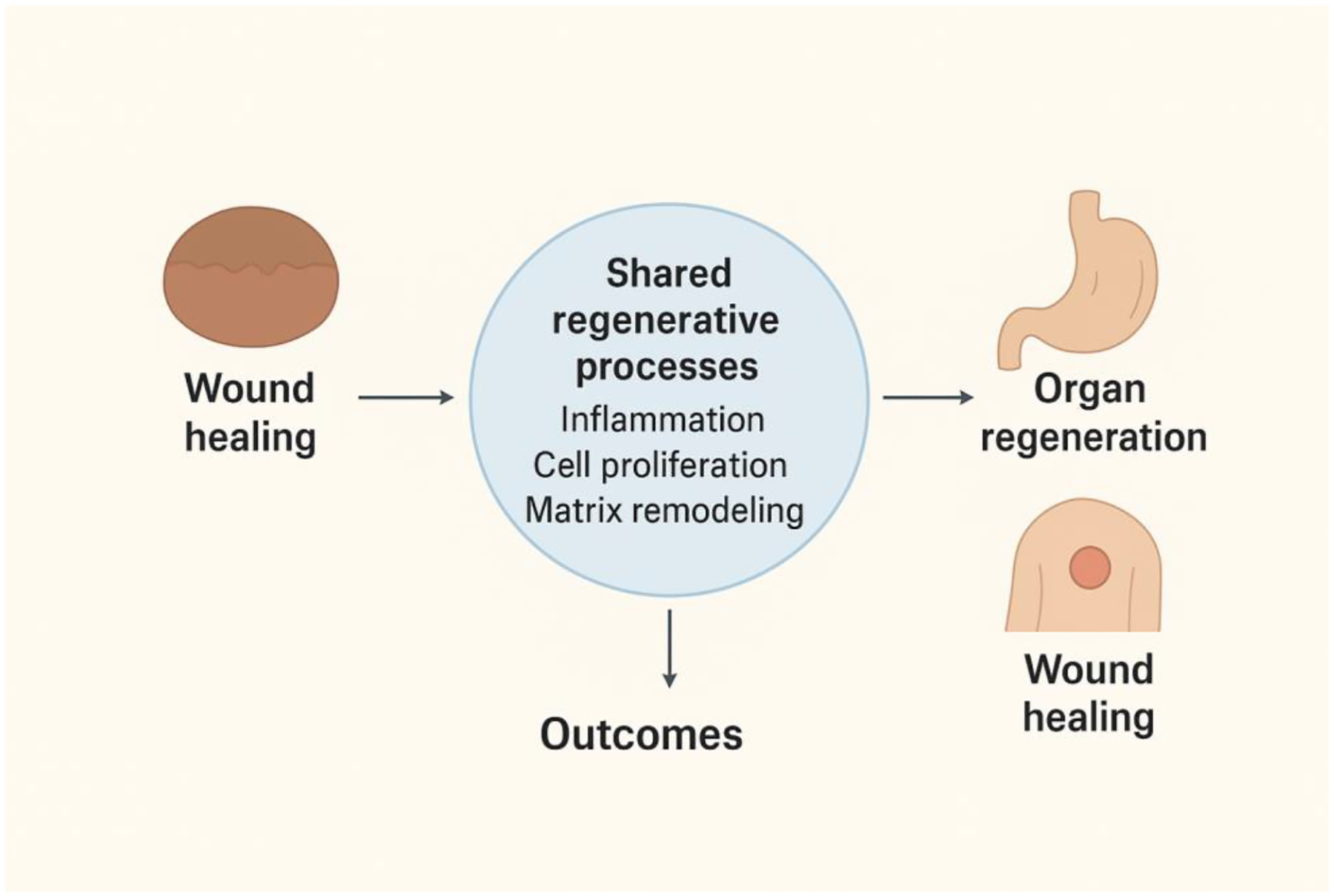
Conceptual diagram illustrating the shared regenerative processes linking wound healing and organ regeneration through coordinated inflammation, cellular proliferation, and extracellular matrix remodeling that drive tissue repair and functional recovery.

While musculoskeletal and hair follicle systems share fundamental regenerative pathways involving stem cell activation, angiogenesis, and immunomodulation driven by exosome cargo, they differ in the specific cell types targeted such as osteoblasts and chondrocytes versus dermal papilla cells and the microenvironmental requirements for tissue specific repair (Figure 2). Engineering exosomes for targeted delivery and controlled release offers a promising approach to address these differences and optimize therapeutic outcomes [24], [27].

## V. Low-Level Laser Therapy

Low-level laser therapy also termed photobiomodulation (PBM), produces regenerative effects through the absorption of red and near-infrared light by cellular chromophores, primarily cytochrome c oxidase in mitochondria. This interaction enhances ATP production, modulates reactive oxygen species, and activates intracellular signaling pathways, resulting in increased cell proliferation, migration, and differentiation, along with reduced inflammation and oxidative stress [32–35]. These mechanisms underpin tissue repair and regeneration across multiple organ systems.

In musculoskeletal healing, LLLT has proven effective in promoting bone and tendon repair, angiogenesis, and osteogenic differentiation of stem cells. The therapy modulates gene expression of growth factors including VEGF, FGF, and TGF-β, and influences the phases of tendon healing by promoting angiogenesis, fibroblast proliferation, and M2 macrophage activation, ultimately enhancing tissue remodeling while reducing inflammation [36–39]. Clinical evidence supports its use for pain reduction and functional improvement in conditions such as osteoarthritis, tendinopathies, and post-surgical recovery.

For hair growth, LLLT has received FDA clearance for androgenetic alopecia and demonstrated safety and efficacy in randomized controlled trials for both male and female pattern hair loss. The mechanisms involve stimulation of hair follicle stem cells, upregulation of growth factors, and modulation of the hair cycle, particularly prolonging the anagen phase [1], [40], [41]. LLLT also reduces follicular inflammation and oxidative stress, contributing to improved hair density and thickness.

Both musculoskeletal and hair follicle regeneration share therapeutic pathways involving activation of mitochondrial metabolism, upregulation of growth factors and modulation of inflammatory and paracrine signaling. Both tissues benefit from enhanced stem cell proliferation and differentiation, angiogenesis, and extracellular matrix remodeling [40]. MicroRNAs and paracrine factors are increasingly recognized as mediators of these effects, with LLLT influencing their expression to promote regenerative outcomes [38].

While musculoskeletal healing and hair follicle restoration share fundamental pathways involving mitochondrial activation, growth factor signaling, and stem cell modulation, tissue-specific differences emerge. Musculoskeletal tissues depend more heavily on collagen synthesis, matrix remodeling, and macrophage polarization, whereas hair follicle restoration centers on hair cycle regulation and follicular stem cell activation [36]. The overlap in microRNA and paracrine signaling reveals a convergent regenerative axis, yet tissue-specific responses to LLLT are determined by local cellular context and repair demands [33].

## VI. Mechanotransduction and Biophysical Signaling

Mechanotransduction is the process by which cells convert mechanical stimuli into biochemical signals, influencing gene expression, cellular behavior, and tissue adaptation. This process involves the sensing of forces such as tension, compression, and shear by cellular structures (including integrins, ion channels, and the cytoskeleton), which trigger intracellular signaling cascades that regulate proliferation, differentiation, and matrix remodeling [42–43]. This fundamental principle underlies both homeostatic maintenance and regenerative responses across diverse tissues.

In musculoskeletal healing, mechanotransduction is essential to tissue repair and remodeling. Mechanical loading through exercise, physical therapy, or targeted rehabilitation activates mechanotransductive pathways that stimulate anabolic responses in bone, tendon, muscle, and cartilage. Loading induces fluid flow and strain within bone, activating osteocytes and promoting bone formation via Wnt signaling and related molecular pathways [44]. Physical therapy frameworks utilize these principles by prescribing specific movement and loading regimens to optimize tissue regeneration and functional recovery, a concept known as “mechanotherapy” [45–47]. Integrating mechanical stimuli with regenerative medicine approaches (such as stem cell therapies and biomaterial scaffolds) further enhances musculoskeletal healing by modulating the local microenvironment and cellular responses [44].

Mechanotransduction also influences hair follicle restoration. Techniques such as microneedling and negative pressure therapy induce controlled micro-injury or mechanical stress in the scalp, triggering the release of growth factors, promoting angiogenesis, and stimulating stem cell activation within the hair follicle niche.[8–9] Wound-induced hair neogenesis demonstrates how mechanical disruption of the skin can lead to de novo hair follicle formation through biophysical signaling and regenerative cascades resembling those in musculoskeletal tissues. These approaches are frequently combined with regenerative therapies (such as PRP and exosomes) to amplify follicular regeneration [5].

At the pathway level, mechanical signaling in musculoskeletal and hair follicle systems shows significant parallels. Both depend on the interplay between extracellular matrix mechanics, stem cell activation, and growth factor release to drive tissue regeneration [43]. The spatiotemporal control of mechanical cues proves critical in both contexts, influencing stem cell fate decisions and orchestrating coordinated tissue remodeling [46]. Physical medicine and rehabilitation thus serve as a bridge, applying mechanotransductive principles to optimize outcomes in both musculoskeletal and hair restoration therapies.

## VII. Clinical Implications

Treatment algorithms vary considerably in their standardization and evidence base. In musculoskeletal medicine, PRP is primarily applied to tendinopathies and osteoarthritis, often as an adjunct to rehabilitation protocols, with dosing and preparation guided by consensus statements such as those from the American Medical Society for Sports Medicine [17]. In hair restoration, PRP, exosomes, and LLLT are used for androgenetic alopecia and alopecia areata, but protocols remain less standardized, showing significant variability in preparation, dosing, and frequency [20], [48], [49]. This lack of uniformity across both fields complicates direct comparison and emphasizes the need for standardized protocols and quality control measures, such as the WESS-PQR scoring system for PRP [48].

Patient response predictors include tissue type, disease chronicity, and individual biological factors. Higher initial platelet counts and proper temperature control during PRP preparation correlate with improved efficacy across tissues. In musculoskeletal applications, factors such as age, comorbidities, and injury chronicity affect outcomes [6]. For hair restoration, predictors include the type of alopecia, follicular density, and scalp vascularity [2–3]. This heterogeneity in response highlights the importance of careful patient selection and individualized treatment planning.

Safety, regulatory, and ethical considerations are particularly relevant in California. PRP and related biologics are generally safe, with minimal adverse effects reported, but preparation heterogeneity and lack of standardization introduce potential risks [48]. Regulatory oversight in California requires compliance with state and federal guidelines for biologic therapies, including documentation of preparation methods and informed consent. Ethical considerations encompass transparency in advertising, avoidance of unproven claims, and equitable access to care. Robust regulatory frameworks and standardized reporting are necessary to ensure patient safety and treatment efficacy [48].

## VIII. Gaps in Knowledge and Future Directions

Despite promising results, significant gaps in knowledge remain. There is a pressing need for standardized biologic preparation and dosing. PRP and exosome therapies suffer from heterogeneity in composition and lack consensus on optimal protocols, which impedes reproducibility and comparability across studies.[20], [50] Comparative trials directly evaluating these modalities in both musculoskeletal and hair applications are scarce, limiting the ability to generalize findings and optimize treatment selection [3]. Additionally, most studies focus on symptom modification rather than true tissue regeneration, and sentinel markers of biologic potency remain poorly defined [51].

Future research should prioritize the development of standardized protocols for biologic therapies, robust comparative effectiveness trials, and the integration of emerging technologies such as gene therapy and advanced cell-based treatments. Research opportunities include elucidating the specific contributions of extracellular vesicles, optimizing mechanotransduction based interventions, and leveraging precision medicine approaches to match biologic treatments to individual patient needs [10]. Addressing these gaps will be essential for advancing regenerative medicine in both physical medicine and rehabilitation and dermatology.

## IX. Conclusion

Regenerative medicine offers a unifying framework for treating both musculoskeletal disorders and hair follicle dysfunction by leveraging shared biological mechanisms that drive tissue repair. Across PRP, exosomes, low level laser therapy, and mechanotransduction, a consistent theme emerges: these modalities stimulate stem cell activity, enhance angiogenesis, modulate inflammation, and promote extracellular matrix remodeling. Despite the distinct structural and functional demands of musculoskeletal and follicular tissues, both systems rely on overlapping molecular pathways including Wnt/β catenin, TGF β, IGF 1, and PDGF to orchestrate regeneration. This convergence underscores the unique position of physical medicine and rehabilitation to integrate regenerative strategies that span orthopedic and dermatologic applications.

At the same time, meaningful challenges persist. Preparation variability in PRP and exosomes, inconsistent dosing protocols, and limited comparative clinical trials hinder widespread standardization and the ability to generalize outcomes across patient populations. Mechanical and photobiomodulatory therapies show robust promise, yet their optimal parameters remain incompletely defined. Regulatory considerations, particularly in California, further highlight the need for transparent reporting, ethical application, and rigorous quality control.

Future progress will depend on harmonizing biologic preparation methods, advancing precision-based treatment algorithms, and developing biomarkers that accurately reflect regenerative potency. Integrating mechanotransductive insights, engineered extracellular vesicles, and emerging gene-based therapies holds potential to elevate both musculoskeletal rehabilitation and hair restoration from symptom focused care to true tissue regeneration. By bridging these traditionally separate domains, regenerative medicine positions itself to transform clinical practice through biologically informed, minimally invasive, and patient centered therapeutic innovation.

## Key points

- Musculoskeletal tissues and hair follicles share core regenerative pathways involving stem cell activation, angiogenesis, inflammatory modulation, and extracellular matrix remodeling.
- Platelet-rich plasma promotes repair in both systems through growth factor–mediated effects on mesenchymal stem cells and follicular stem cells.
- Exosomes function as critical paracrine mediators, delivering microRNAs and proteins that regulate Wnt/β-catenin, TGF-β, and angiogenic signaling across tissues.
- Low-level laser therapy enhances mitochondrial metabolism, growth factor expression, and stem cell proliferation in both orthopedic rehabilitation and hair restoration.
- Mechanotransduction links physical therapy, exercise, microneedling, and mechanical loading to biologically driven regeneration through biophysical signaling.
- Despite shared pathways, tissue-specific differences exist, with musculoskeletal repair emphasizing matrix remodeling and follicular restoration emphasizing hair cycle regulation.
- Clinical protocols for PRP, exosomes, and LLLT remain heterogeneous, limiting reproducibility and comparative effectiveness across studies.
- Physical medicine and rehabilitation provide a unique framework to integrate biologics, photobiomodulation, and mechanotherapy into coordinated regenerative strategies.
- Future progress depends on standardized biologic preparation, precision-based patient selection, and biomarkers that reflect true regenerative potency.

## References

[1] Liu D, Xu Q, Meng X, X. , “Status of research on the development and regeneration of hair follicles,” Int. J. Med. Sci, 21 (2024): 80–94. 38164355 10.7150/ijms.88508 PMC10750333

[2] Semsarzadeh N and Khetarpal S, “Platelet-Rich Plasma and Stem Cells for Hair Growth: A Review of the Literature,” Aesthet. Surg. J, 40 (2020): NP177–NP188. 31111157 10.1093/asj/sjz146

[3] Anudeep TC , “Advancing Regenerative Cellular Therapies in Non-Scarring Alopecia,” Pharmaceutics, 14 (2022): p.612, 35335987 10.3390/pharmaceutics14030612 PMC8953616

[4] Wu W-S, Chen L-R, and Chen K-H, “Platelet-Rich Plasma (PRP): Molecular Mechanisms, Actions and Clinical Applications in Human Body,” Int. J. Mol. Sci, 26 (2025): p.10804. 41226837 10.3390/ijms262110804 PMC12608683

[5] Paichitrojjana A and Paichitrojjana A, “Platelet Rich Plasma and Its Use in Hair Regrowth: A Review,” Drug Des. Devel. Ther, 16 (2022): pp.635–645, 10.2147/DDDT.S356858 PMC8922312 35300222

[6] Everts P, Onishi K, Jayaram P, , “Platelet-Rich Plasma: New Performance Understandings and Therapeutic Considerations in 2020,” Int. J. Mol. Sci, 21 (2020): p.7794. 33096812 10.3390/ijms21207794 PMC7589810

[7] Dos Santos RG , “The regenerative mechanisms of platelet-rich plasma: A review,” Cytokine, 144 (2021): p.155560. 34004552 10.1016/j.cyto.2021.155560

[8] Etulain J, “Platelets in wound healing and regenerative medicine,” Platelets, 29 (2018): pp.556–568. 29442539 10.1080/09537104.2018.1430357

[9] Samadi P, Sheykhhasan M, Khoshinani HM, “The Use of Platelet-Rich Plasma in Aesthetic and Regenerative Medicine: A Comprehensive Review,” Aesthetic Plast. Surg, 43 (2019): pp.803–814. 30552470 10.1007/s00266-018-1293-9

[10] Anitua E, Troya M, Falcon-Pérez JM, , “Advances in Platelet Rich Plasma-Derived Extracellular Vesicles for Regenerative Medicine: A Systematic-Narrative Review,” Int. J. Mol. Sci, vol. 24 (2023): p.13043. 37685849 10.3390/ijms241713043 PMC10488108

[11] Miron RJ, Estrin NE, Sculean A, Zhang Y, “Understanding exosomes: Part 2-Emerging leaders in regenerative medicine,” Periodontol. 2000, 94 (2024): pp.257–414. 10.1111/prd.12561 38591622

[12] Qian Y , “Platelet-Rich Plasma Derived Growth Factors Contribute to Stem Cell Differentiation in Musculoskeletal Regeneration,” Front. Chem, 5 (2017): p.89. 29164105 10.3389/fchem.2017.00089 PMC5671651

[13] Giannotti L , “Progress in Regenerative Medicine: Exploring Autologous Platelet Concentrates and Their Clinical Applications,” Genes, 14 (2023): p.1669. 37761809 10.3390/genes14091669 PMC10530962

[14] Yuan A-R, Bian Q, and Gao J-Q, “Current advances in stem cell-based therapies for hair regeneration,” Eur. J. Pharmacol 881 (2020): p.173197. 32439260 10.1016/j.ejphar.2020.173197

[15] Gentile P and Garcovich S, “Advances in Regenerative Stem Cell Therapy in Androgenic Alopecia and Hair Loss: Wnt pathway, Growth-Factor, and Mesenchymal Stem Cell Signaling Impact Analysis on Cell Growth and Hair Follicle Development,” Cells, 8 (2019): p. 466. 31100937 10.3390/cells8050466 PMC6562814

[16] Cecerska-Heryć E , “Applications of the regenerative capacity of platelets in modern medicine,” Cytokine Growth Factor Rev. 64 (2022): pp. 84–94. 34924312 10.1016/j.cytogfr.2021.11.003

[17] Finnoff JT , “American Medical Society for Sports Medicine Position Statement: Principles for the Responsible Use of Regenerative Medicine in Sports Medicine,” Clin. J. Sport Med, 31 (2021): p.530. 34704973 10.1097/JSM.0000000000000973

[18] Hesseler MJ and Shyam N, “Platelet-Rich Plasma and Its Utilities in Alopecia: A Systematic Review,” Dermatol. Surg. Off. Publ. Am. Soc. Dermatol. Surg. Al, 46 (2020): pp.93–102. 10.1097/DSS.0000000000001965 31211715

[19] Singh M, Harary J, Schilling PL, and Moschetti WE, “Patient Satisfaction Is Nearly 90% After Total Knee Arthroplasty; We Are Better Than We Were,” J. Arthroplasty, (2024): pp.S0883–5403(24)01251–8. 10.1016/j.arth.2024.11.038 39581239

[20] Kourosh AS , “Platelet-Rich Plasma: Advances and Controversies in Hair Restoration and Skin Rejuvenation,” Dermatol. Surg. Off. Publ. Am. Soc. Dermatol. Surg. Al, 50 (2024), pp. 446–452. 10.1097/DSS.0000000000004115 38376068

[21] Zhu M , “Platelet sonicates activate hair follicle stem cells and mediate enhanced hair follicle regeneration,” J. Cell. Mol. Med, 24 (2020): pp.1786–1794. 31802614 10.1111/jcmm.14873 PMC6991668

[22] Cobelli NJ, Leong DJ, and Sun HB, “Exosomes: biology, therapeutic potential, and emerging role in musculoskeletal repair and regeneration,” Ann. N. Y. Acad. Sci, vol. 1410 (2017): pp.57–67. 29125180 10.1111/nyas.13469

[23] Yao X, Wei W, Wang X, L. , “Stem cell derived exosomes: microRNA therapy for age-related musculoskeletal disorders,” Biomaterials, 224 (2019): p.119492. 31557588 10.1016/j.biomaterials.2019.119492

[24] Deng S, Cao H, Cui X, Fan Y, , “Optimization of exosome-based cell-free strategies to enhance endogenous cell functions in tissue regeneration,” Acta Biomater., 171 (2023): pp.68–84. 37730080 10.1016/j.actbio.2023.09.023

[25] Youssef El Baradie KB and Hamrick MW, “Therapeutic application of extracellular vesicles for musculoskeletal repair & regeneration,” Connect. Tissue Res, 62 (2021): pp.99–114. 32602385 10.1080/03008207.2020.1781102

[26] Chen L-Y , “Frontier Review of the Molecular Mechanisms and Current Approaches of Stem Cell-Derived Exosomes,” Cells, 12 (2023): p.1018. 37048091 10.3390/cells12071018 PMC10093591

[27] Lv Z , “Exosome-Based Therapeutics for Musculoskeletal Disorders: Advances in Engineering, Targeting, and Biomaterial Integration,” ACS Nano 19 (2025): 33681–33716. 40963099 10.1021/acsnano.5c05416 PMC12490021

[28] Carrasco E, Soto-Heredero G, and Mittelbrunn M, “The Role of Extracellular Vesicles in Cutaneous Remodeling and Hair Follicle Dynamics,” Int. J. Mol. Sci, vol. 20 (2019): p.2758. 31195626 10.3390/ijms20112758 PMC6600598

[29] Zhou Y, Seo J, Tu S, Nanmo A, , “Exosomes for hair growth and regeneration,” J. Biosci. Bioeng, 137 (2024): pp.1–8. 37996318 10.1016/j.jbiosc.2023.11.001

[30] Queen D and Avram MR, “Exosomes for Treating Hair Loss: A Review of Clinical Studies,” Dermatol. Surg. Off. Publ. Am. Soc. Dermatol. Surg. Al, 51 (2025): pp.409–415. 10.1097/DSS.0000000000004480 39447204

[31] da Costa Pereira Cestari M, Falavigna Tovo R, and Franco Bueno D, “MSC-Derived Secretome and Exosomes in Dermatology: Mechanisms, Therapeutic Opportunities, and Scientific Challenges-A Narrative Review,” Int. J. Dermatol, Aug. (2025). 10.1111/ijd.17982 PMC12783422 40751329

[32] Dompe C , “Photobiomodulation-Underlying Mechanism and Clinical Applications,” J. Clin. Med, 9 (2020): p.1724. 32503238 10.3390/jcm9061724 PMC7356229

[33] Tamimi R, Benisi SZ, Boroujeni ME, , “Review on the molecular mechanisms of low-level laser therapy: gene expression and signaling pathways,” Lasers Med. Sci, 40 (2025): p.160. 40131476 10.1007/s10103-025-04393-z

[34] Glass GE, “Photobiomodulation: The Clinical Applications of Low-Level Light Therapy,” Aesthet. Surg. J, 41 (2021): pp.723–738. 33471046 10.1093/asj/sjab025

[35] Hernández-Bule ML, Naharro-Rodríguez J, Bacci S, , “Unlocking the Power of Light on the Skin: A Comprehensive Review on Photobiomodulation,” Int. J. Mol. Sci, vol. 25 (2024): p.4483. 38674067 10.3390/ijms25084483 PMC11049838

[36] Berni M , “The Role of Low-Level Laser Therapy in Bone Healing: Systematic Review,” Int. J. Mol. Sci, 24 (2023): p.7094. 37108257 10.3390/ijms24087094 PMC10139216

[37] Lyu K , “The Functions and Mechanisms of Low-Level Laser Therapy in Tendon Repair (Review),” Front. Physiol, 13 (2022): p.808374. 35242050 10.3389/fphys.2022.808374 PMC8886125

[38] Illescas-Montes R , “Human Fibroblast Gene Expression Modulation Using 940 NM Diode Laser,” Sci. Rep, 9 (2019): p.12037. 31427686 10.1038/s41598-019-48595-2 PMC6700136

[39] DE Oliveira MF, Johnson DS, Demchak T, , “Low-intensity LASER and LED (photobiomodulation therapy) for pain control of the most common musculoskeletal conditions,” Eur. J. Phys. Rehabil. Med, 58 (2022): pp.282–289. 34913330 10.23736/S1973-9087.21.07236-1 PMC9980499

[40] Zarei M, Wikramanayake TC, Falto-Aizpurua L, Schachner LA, and Jimenez JJ, “Low level laser therapy and hair regrowth: an evidence-based review,” Lasers Med. Sci, 31 (2016): pp.363–371. 26690359 10.1007/s10103-015-1818-2

[41] Ring CM, Finney R, and Avram M, “Lasers, lights, and compounds for hair loss in aesthetics,” Clin. Dermatol, 40 (2022): pp.64–75. 35190067 10.1016/j.clindermatol.2021.08.013

[42] Dunn SL and Olmedo ML, “Mechanotransduction: Relevance to Physical Therapist Practice-Understanding Our Ability to Affect Genetic Expression Through Mechanical Forces,” Phys. Ther, 96 (2016): pp.712–721. 26700270 10.2522/ptj.20150073

[43] Bakhshandeh B , “Mechanotransduction in tissue engineering: Insights into the interaction of stem cells with biomechanical cues,” Exp. Cell Res, 431 (2023): p.113766. 37678504 10.1016/j.yexcr.2023.113766

[44] Glatt V, Evans CH, and Stoddart MJ, “Regenerative rehabilitation: The role of mechanotransduction in orthopaedic regenerative medicine,” J. Orthop. Res. Off. Publ. Orthop. Res. Soc, 37 (2019): pp.1263–1269. 10.1002/jor.24205 PMC6546504 30561813

[45] Thompson WR, Scott A, Loghmani MT, , “Understanding Mechanobiology: Physical Therapists as a Force in Mechanotherapy and Musculoskeletal Regenerative Rehabilitation,” Phys. Ther, 96 (2016): pp.560–569. 26637643 10.2522/ptj.20150224 PMC4817213

[46] Ng JL, Kersh ME, Kilbreath S, , “Establishing the Basis for Mechanobiology-Based Physical Therapy Protocols to Potentiate Cellular Healing and Tissue Regeneration,” Front. Physiol, vol. 8 (2017): p.303. 28634452 10.3389/fphys.2017.00303 PMC5460618

[47] Khan MA , “A systematic review on functional electrical stimulation based rehabilitation systems for upper limb post-stroke recovery,” Front. Neurol, 14 (2023): p.1272992. 38145118 10.3389/fneur.2023.1272992 PMC10739305

[48] Rahman E , “Systematic Review of Platelet-Rich Plasma in Medical and Surgical Specialties: Quality, Evaluation, Evidence, and Enforcement,” J. Clin. Med, 13 (2024): p.4571. 39124838 10.3390/jcm13154571 PMC11313071

[49] Popescu MN , “Autologous Platelet-Rich Plasma Efficacy in the Field of Regenerative Medicine: Product and Quality Control,” BioMed Res. Int, (2021): p.4672959. 34368346 10.1155/2021/4672959 PMC8346315

[50] Gupta AK, Renaud HJ, and Rapaport JA, “Platelet-rich Plasma and Cell Therapy: The New Horizon in Hair Loss Treatment,” Dermatol. Clin, 39 (2021): pp.429–445. 34053596 10.1016/j.det.2021.04.001

[51] Rodeo SA, “Orthobiologics: Current Status in 2023 and Future Outlook,” J. Am. Acad. Orthop. Surg, vol. 31 (2023): pp.604–613. 37130369 10.5435/JAAOS-D-22-00808

